# Is insulin the preferred treatment in persons with type 2 diabetes and liver cirrhosis?

**DOI:** 10.1186/s12876-021-01773-x

**Published:** 2021-06-12

**Authors:** Fu-Shun Yen, Jung-Nien Lai, James Cheng-Chung Wei, Lu-Ting Chiu, Chih-Cheng Hsu, Ming-Chih Hou, Chii-Min Hwu

**Affiliations:** 1 Dr. Yen’s Clinic, No. 15, Shanying Road, Gueishan District, Taoyuan, 33354 Taiwan; 2 School of Chinese Medicine, College of Chinese Medicine, China Medical University, No. 91, Hsueh-Shih Road, Taichung, 40402 Taiwan; 3 Department of Chinese Medicine, China Medical University Hospital, 3F., No. 373-2, Jianxing Road, Taichung, 40459 Taiwan; 4 Department of Allergy, Immunology and Rheumatology, Chung Shan Medical University Hospital, Taichung City, 40201 Taiwan; 5 Institute of Medicine, Chung Shan Medical University, Taichung City, 40201 Taiwan; 6 Graduate Institute of Integrated Medicine, China Medical University, No. 110, Sec. 1, Jianguo N. Rd., South District, Taichung City, 40201 Taiwan; 7 Management Office for Health Data, China Medical University Hospital, 3F., No. 373-2, Jianxing Road, Taichung, 40459 Taiwan; 8 College of Medicine, China Medical University, No. 110, Sec. 1, Jianguo N. Rd., South District, Taichung City, 40201 Taiwan; 9 Institute of Population Health Sciences, National Health Research Institutes, 35 Keyan Road, Zhunan, Miaoli County 35053 Taiwan; 10 Department of Health Services Administration, China Medical University, No. 91, Hsueh-Shih Road, Taichung, 40402 Taiwan; 11 Department of Family Medicine, Min-Sheng General Hospital, 168 ChingKuo Road, Taoyuan, 33044 Taiwan; 12 Faculty of Medicine, National Yang-Ming University School of Medicine, No. 155, Sec. 2, Linong Street, Taipei, 11221 Taiwan; 13 Division of Gastroenterology and Hepatology, Department of Medicine, Taipei Veterans General Hospital, No. 201, Sec. 2, Shipai Road, Beitou District, Taipei, 11217 Taiwan; 14 Section of Endocrinology and Metabolism, Department of Medicine, Taipei Veterans General Hospital, No. 201, Sec. 2, Shipai Road, Beitou District, Taipei, 11217 Taiwan

**Keywords:** All-cause mortality, Hepatocellular carcinoma, Hepatic failure, Decompensated cirrhosis, Hypoglycemia

## Abstract

**Background:**

Insulin is highly recommended for diabetes management in persons with liver cirrhosis. However, few studies have evaluated its long-term effects in these persons. We conducted this study to compare the risks of mortality, liver-related complications, and cardiovascular events in persons with type 2 diabetes mellitus (T2DM) and compensated liver cirrhosis.

**Methods:**

From January 1, 2000, to December 31, 2012, we selected 2047 insulin users and 4094 propensity score-matched nonusers from Taiwan’s National Health Insurance Research Database. Cox proportional hazard models were used to assess the risks of outcomes.

**Results:**

The mean follow-up time was 5.84 years. The death rate during the follow-up period was 5.28 and 4.07 per 100 person-years for insulin users and nonusers, respectively. In insulin users, the hazard ratios and 95% confidence intervals (CIs) of all-cause mortality, hepatocellular carcinoma, decompensated cirrhosis, hepatic failure, major cardiovascular events, and hypoglycemia were 1.31 (1.18–1.45), 1.18 (1.05–1.34), 1.53 (1.35–1.72), 1.26 (1.42–1.86), 1.41 (1.23–1.62), and 3.33 (2.45–4.53), respectively.

**Conclusions:**

This retrospective cohort study indicated that among persons with T2DM and compensated liver cirrhosis, insulin users were associated with higher risks of death, liver-related complications, cardiovascular events, and hypoglycemia compared with insulin nonusers.

## Background

Insulin has saved numerous lives since its discovery in the 1920s. It is extremely effective in treating hyperglycemia and can be used when hyperglycemia causes critical conditions, such as ketoacidosis or hyperosmolar hyperglycemic state [1]. By carefully manipulating the dose, insulin is also frequently used in persons with hospitalization, major surgery, sepsis, and acute myocardial infarction [2]. Liver cirrhosis also is the strong indication for insulin treatment in persons with type 2 diabetes mellitus (T2DM) [1, 2].

Liver cirrhosis is an advanced liver disease; it also is the late stage of chronic liver injury [3]. It can be attributed to several reasons, such as nonalcoholic fatty liver diseases, chronic alcoholism, hepatitis B virus (HBV) infection, or hepatitis C virus (HCV) infection [3]. With the development of cirrhosis, owing to reduced insulin extraction of liver and portal-systemic shunting, serum insulin levels will increase and insulin resistance may develop. Approximately 96% of persons with cirrhosis may be glucose intolerant and 30% of them may develop clinical diabetes [4]. Moreover, diabetes treatment in persons with liver cirrhosis is complex [5]. Diet control for persons with cirrhosis is not feasible because they may have poor appetite. Encouraging them to exercise may not be suitable because they may demonstrate weakness. Medications such as metformin, sulphonylureas, and thiazolidinedione may cause lactic acidosis (especially in those with chronic alcoholism), may lead to the risk of hypoglycemia, and may aggravate fluid retention, respectively. Thus, adequate management of T2DM in persons with liver cirrhosis is unclear.

Careful adjustment of the insulin dose and close monitoring of blood glucose levels may enable the effective and safe use of insulin for treating persons with cirrhosis and T2DM [6]. However, insulin has some deleterious side effects. Hypoglycemia is the most critical side effect of insulin use, as it can increase the risks of mortality and cardiovascular diseases [7]. Insulin was also reported to increase body weight and risks of cardiovascular events [8, 9], and insulin use is associated with the risk of hepatocellular carcinoma (HCC) [10]. Therefore, we conducted this retrospective cohort study to investigate the long-term outcomes of insulin use in people with T2DM and compensated liver cirrhosis.

## Methods

### Participants

This study recruited persons with new diagnoses of T2DM and liver cirrhosis from the Longitudinal Cohort of Diabetes Patients (LHDB) between January 1, 2000, and December 31, 2012, and they were followed until December 31, 2013. LHDB is part of the National Health Insurance (NHI) Research Database (NHIRD). It comprises data of 1,700,000 randomly selected newly diagnosed T2DM patients with longitudinally linked data available from 1997 to 2013. The NHIRD includes the health records of insured persons in Taiwan’s NHI program, which was established in 1995 and covered approximately 99% of Taiwan’s 23 million people by 2000 [11]. This administrative database contains information of age, birth date, sex, living areas, treatments, and disease diagnoses according to International Classification of Diseases, Ninth Revision, Clinical Modification (ICD-9-CM) codes. This study was conducted in accordance with the Declaration of Helsinki. To protect personal privacy, all information on the care providers or patients was scrambled before being released. This study was approved by the Research Ethics Committee of China Medical University and Hospital (CMUH104-REC2-115), and the need for informed consent was waived.

### Study design

T2DM was diagnosed based on ICD-9-CM code 250.xx with at least 2 outpatient claims within 1 year or one hospitalization. Persons with ICD-9-CM code 571.5, 571.2, or 571.6 for at least 2 outpatient claims within 1 year or one hospitalization were defined as having liver cirrhosis. This method of defining T2DM and liver cirrhosis using ICD-9-CM codes has been validated by studies [12, 13]; the diagnostic accuracy of diabetes and cirrhosis is 74.6% and 82.6%, respectively. Persons with liver cirrhosis and esophageal varices with bleeding (456.0 and 456.2), ascites (789.59 and 789.5), hepatic encephalopathy (572.2), or jaundice (782.4) were defined as having decompensated liver cirrhosis [14] and were initially excluded from this study. Patients without these cirrhotic complications were defined as having compensated liver cirrhosis. We excluded persons who were diagnosed as having type 1 diabetes mellitus (ICD-9-CM code 250.1); who did not receive antidiabetic medications; who were younger than 18 years or older than 80 years; who lacked gender information; who died or had renal failure, stroke, ischemic heart disease, heart failure, HCC, esophageal varices with bleeding, ascites, hepatic encephalopathy, jaundice, or hepatic failure before the index date or within 6 months after the index date; and who were diagnosed as having T2DM or cirrhosis during 1997–1999.

### Procedures

The day of concomitant diagnosis of liver cirrhosis and diabetes was defined as the comorbid date. Persons who underwent insulin therapy for at least 28 days after the comorbid date were defined as insulin users, and those who never took insulin during the whole study period were defined as insulin nonusers. We defined the first date of insulin use as the index date. Variables considered as potential confounders in this study included age, sex, age at the diagnosis of diabetes, the duration of diabetes, antihypertensive and antidiabetic medications, statins, and aspirin. Comorbidities status before the index date included HCV (ICD-9-CM codes 070.41, 070.44, 070.51, 070.54, 070.70, 070.71, and V02.62) and HBV infections (ICD-9-CM codes 070.2, 070.3, and V02.61). We also calculated the Charlson comorbidity index (CCI; a weighted index that consider the number and seriousness of comorbid heart, vascular, chronic pulmonary, connective tissue, mild and moderate liver, ulcer disease, diabetes and related complication, any original or metastatic disease, and AIDS) [15] and Diabetes Complication Severity Index (DCSI; including cardiovascular disease, nephropathy, retinopathy, peripheral vascular disease, stroke, neuropathy, and metabolic diabetes complications) scores [16] to assess the severity of diabetes.

### Main outcomes

We investigated the outcomes of all-cause mortality, major adverse cardiovascular events (MACE), HCC, decompensated cirrhosis, hepatic failure, and hypoglycemia. Death was defined as being discharged from the hospital with a death certificate (discharge date was defined as the death date) or termination of NHI coverage after being discharged from hospital due to a critical illness and no further healthcare use for more than 1 year (the end of NHI coverage was defined as the death date). We calculated the incidence rate of MACE, including ischemic heart disease (410–414), stroke (430–437), and heart failure (428); HCC (155.x); decompensated cirrhosis (the composite of esophageal varices with bleeding, ascites, hepatic encephalopathy, and jaundice); variceal bleeding; ascites; hepatic encephalopathy; and hepatic failure (570, 572.2, 572.4, and 572.8), to evaluate liver-related complications. We also investigated the incidence of emergency department visited or admitted hypoglycemia (251.0x, 251.1x, or 251.2x) to evaluate the probable complications of treatments.

### Statistical analyses

Propensity score matching was adopted to optimize comparability between insulin users and nonusers [17]. The propensity score was estimated for every person through nonparsimonious multivariable logistic regression, with insulin treatment as the dependent variable. We used 26 clinically related variables in the analysis as controlling variables (Table 1). The nearest-neighbor algorithm was adopted to construct matching pairs under the assumption that the proportion of 0.995–1.0 was perfect [18].

**Table 1:** Baseline characteristics of insulin users and nonusers with diabetes and compensated liver cirrhosis

Variables	Before propensity score match					After propensity score match				
	Non-insulin users (n = 17,173)		Insulin users (n = 2047)		*p* value	Non-insulin users (n = 4094)		Insulin users (n = 2047)		*p* value
	N	%	N	%		n	%	n	%	
Age					0.01					0.54
18–49	5886	34.27	732	35.76		1444	35.27	732	35.76	
50–65	7301	42.51	900	43.97		1793	43.80	900	43.97	
> 65	3986	23.22	415	20.27		857	20.93	415	20.27	
Mean ± SD	55.37 ± 11.91		54.98 ± 11.32		0.15	55.20 ± 11.82		54.98 ± 11.32		0.29
Sex					0.11					0.62
Female	5421	31.57	611	29.85		1197	29.24	611	29.85	
Male	11,752	68.43	1436	70.15		2897	70.76	1436	70.15	
DM age, mean ± SD	55.96 ± 11.12		51.93 ± 10.93		< 0.0001	52.58 ± 11.03		51.93 ± 10.93		0.06
DM duration, mean ± SD^a^	4.85 ± 3.47		3.05 ± 2.79		< 0.0001	3.21 ± 3.94		3.05 ± 2.79		0.10
Antihypertensive drugs										
ACEI/ARB	7401	43.10	1076	52.56	< 0.0001	2143	52.34	1076	52.56	0.87
β-blockers	9004	52.43	1298	63.41	< 0.0001	2567	62.70	1298	63.41	0.59
Calcium-channel blockers	5086	29.62	777	37.96	< 0.0001	1495	36.52	777	37.96	0.27
Diuretics	4304	25.06	699	34.15	< 0.0001	1384	33.81	699	34.15	0.79
Other anti-hypertensive agent	3350	19.51	521	25.45	< 0.0001	1034	25.26	521	25.45	0.87
Antidiabetic drugs										
Metformin	5437	31.66	1033	50.46	< 0.0001	2036	49.73	1033	50.46	0.59
Sulfonylurea	6446	37.54	1178	57.55	< 0.0001	2337	57.08	1178	57.55	0.73
Meglitinide	1814	10.56	327	15.97	< 0.0001	644	15.73	327	15.97	0.80
Thiazolidinedione	2050	11.94	429	20.96	< 0.0001	789	19.27	429	20.96	0.12
α-glucosidase inhibitor	1779	10.36	341	16.66	< 0.0001	644	15.73	341	16.66	0.35
DPP-4 inhibitors	457	2.66	92	4.49	< 0.0001	199	4.86	92	4.49	0.53
Other drugs										
Statin	4202	24.47	661	32.29	< 0.0001	1299	31.73	661	32.29	0.66
Aspirin	9556	55.65	1354	66.15	< 0.0001	2762	67.46	1354	66.15	0.30
DCSI score					< 0.0001					0.59
0	9298	54.14	732	35.76		1418	34.64	732	35.76	
1	2595	15.11	397	19.39		786	19.20	397	19.39	
≥ 2	5280	30.75	918	44.85		1890	46.17	918	44.85	
CCI					< 0.0001					0.64
0	11,657	67.88	694	33.90		1357	33.15	694	33.90	
1	2326	13.54	528	25.79		1101	26.89	528	25.79	
≥ 2	3190	18.58	825	40.31	< 0.0001	1636	39.96	825	40.30	
HBV	2810	16.36	491	23.99	< 0.0001	928	22.67	491	23.99	0.25
HCV	2093	12.19	407	19.88	< 0.0001	804	19.64	407	19.88	0.82

SD, standard deviation; ACEI, angiotensin converting enzyme inhibitor; ARB, angiotensin receptor blocker; CCI, Charlson comorbidity index; DCSI score, diabetes complications severity index score; HBV, hepatitis B virus; HCV, hepatitis C virus

^a^*t*-test

Crude and multivariate-adjusted Cox proportional hazard models with robust sandwich standard error estimates were used to compare the risk of outcomes between insulin users and nonusers. The results are presented as hazard ratios (HRs) with 95% confidence intervals (CIs) for insulin users versus nonusers. To assess the risk of all-cause mortality, we checked the persons’ time of death or the end of the study, whichever occurred first. For other outcomes, we checked the persons’ date of respective outcomes or end of follow-up on December 31, 2013, whichever occurred first. We compared the cumulative incidence of all-cause mortality, MACE, decompensated cirrhosis, and hepatic failure over time between insulin users and nonusers using the Kaplan–Meier method and log-rank tests.

We conducted a sensitivity test by excluding persons with hypoglycemia before or after the index date; matching insulin users and nonusers; and calculating the incidence and hazard ratio of death, MACE, and liver-related outcomes to avoid the interference from hypoglycemia on other main outcomes.

Two-tailed *P* < 0.05 was considered as significant. SAS v9.4 (SAS Institute, Inc., Cary, NC, USA) was used for the analyses.

## Results

### Participants

**Fig. 1:**
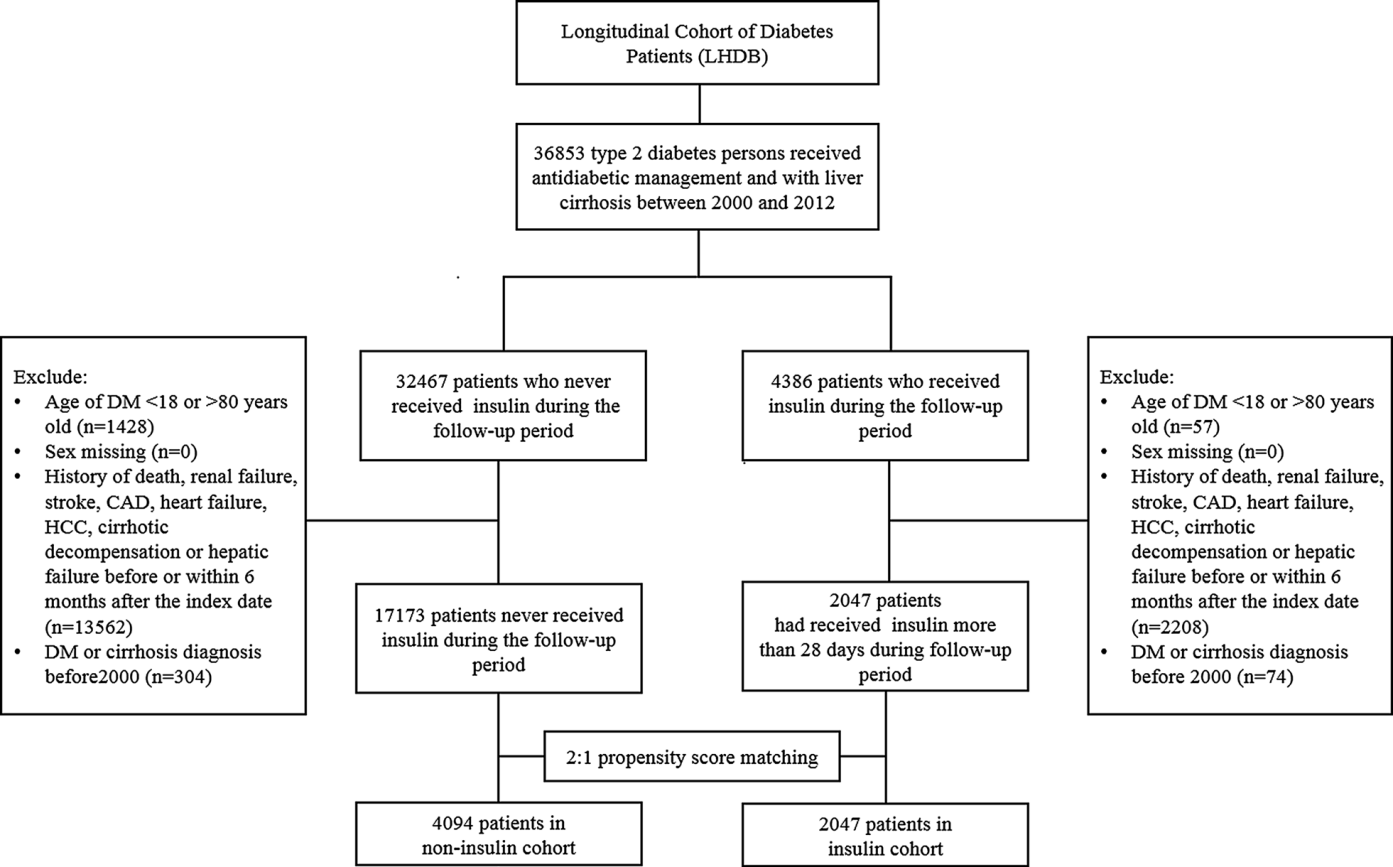
Flowchart of patient selection for this study

From January 1, 2000, to December 31, 2012, a total of 36,853 persons were diagnosed as having T2DM with compensated cirrhosis and were undergoing anti-diabetes treatment. After exclusion of ineligible cases, 2047 persons received insulin treatment for at least 28 days, and 17,173 persons had never received insulin during the follow-up period. Figure 1 depicts the flowchart of patient selection for this study.

Before matching, insulin users presented higher proportions of young age, young ages at diabetes diagnosis, medications use, hepatitis infection than insulin nonusers. People with high CCI and DCSI scores were found among insulin users than among insulin nonusers (Table 1). After propensity score matching, 2047 insulin users and 4094 insulin nonusers were selected. Of insulin users, 1650 (80.61%), 1289 (62.97%), and 1781 (87.01%) persons used basal insulin, premixed insulin, and prandial insulin, respectively. The matched patients are similar in all variables. The mean age of this cohort was 55.09 years, the mean duration of diabetes was 3.13 years, and the HBV and HCV infection rates were 23.33% and 19.76%, respectively. The mean follow-up time was 5.79 years for insulin users and 5.88 years for nonusers.

### Risks of outcomes

In the matched cohort of people with T2DM and compensated liver cirrhosis, 627 (30.63%) insulin users and 979 (23.91%) insulin nonusers died during the follow-up period (incidence rate of 5.28 vs 4.07 per 100 patient-years, respectively). The multivariable-adjusted HR (95% CI) of insulin users to nonusers was 1.31 (1.18–1.45; Table 2).

**Table 2:** Outcomes of insulin users and matched nonusers with diabetes and compensated liver cirrhosis

Outcomes	Non-insulin users (n = 4094)			Insulin users (n = 2047)			Crude HR (95% CI)	*p* value	Adjusted HR^a^ (95% CI)	*p* value
	Events	PY	IR	Events	PY	IR				
All-cause mortality	979	24,075	4.07	627	11,866	5.28	1.33 (1.21˗1.48)	< 0.0001	1.31 (1.18˗1.45)	< 0.0001
HCC	700	22,490	3.11	402	10,921	3.68	1.17 (1.03˗1.32)	0.01	1.18 (1.05˗1.34)	0.007
MACE	512	22,227	2.30	339	10,738	3.16	1.37 (1.19˗1.57)	< 0.0001	1.41 (1.23˗1.62)	< 0.0001
Stroke	300	23,086	1.30	186	11,285	1.65	1.28 (1.07˗1.54)	0.007	1.31 (1.09˗1.58)	0.004
Ischemic heart disease	198	23,277	0.85	128	11,394	1.12	1.31 (1.05˗1.64)	0.02	1.36 (1.09˗1.71)	0.006
Heart failure	125	23,649	0.53	128	11,507	1.11	2.11 (1.65˗2.71)	< 0.0001	2.18 (1.70˗2.80)	< 0.0001
Decompensated cirrhosis	642	22,865	2.81	465	10,890	4.27	1.50 (1.33˗1.69)	< 0.0001	1.53 (1.35˗1.72)	< 0.0001
Variceal bleeding	41	23,986	0.17	38	11,767	0.32	1.83 (1.18˗2.85)	0.007	1.81 (1.16˗2.83)	0.009
Hepatic ascites	407	23,381	1.74	327	11,196	2.92	1.66 (1.43˗1.92)	< 0.0001	1.68 (1.45˗1.95)	< 0.0001
Hepatic encephalopathy	351	23,577	1.49	281	11,380	2.47	1.64 (1.40˗1.91)	< 0.0001	1.63 (1.39˗1.91)	< 0.0001
Jaundice	102	23,848	0.43	45	11,757	0.38	0.88 (0.62˗1.25)	0.49	0.90 (0.63˗1.29)	0.58
Hepatic failure	493	23,363	2.11	388	11,230	3.46	1.62 (1.42˗1.85)	< 0.0001	1.26 (1.42˗1.86)	< 0.0001
Hypoglycemia	68	23,893	0.28	107	11,576	0.92	3.26 (2.40˗4.42)	< 0.0001	3.33 (2.45˗4.53)	< 0.0001

PY, person-years; IR, incidence rate, per 100 person-years; HR, hazard ratio; CI, confidence interval; HCC, hepatocellular carcinoma; MACE, major adverse cardiac event, including stroke, ischemic heart disease, and heart failure

^a^Adjusted for age, sex, index year, age of diabetes mellitus diagnosis, DM duration (years), antihypertensive drugs (ACE inhibitors, ARBs, β-blockers, calcium-channel blockers, diuretics, other antihypertensive), antidiabetic drugs (metformin, sulfonylureas, meglitinides, TZD, α-glucosidase inhibitor, DPP-4 inhibitors), statin, and aspirin, CCI (0, 1, ≥ 2), DCSI score (0, 1, ≥ 2), HBV and HCV

Table 2 shows that insulin users associated with higher risks of HCC (adjusted hazard ratio [aHR] [95% CI]: 1.18 [1.05–1.34]), decompensated cirrhosis (aHR [95% CI]: 1.53 [1.35–1.72]), esophageal varices with bleeding (aHR [95% CI]: 1.81 [1.16–2.83]), hepatic ascites (aHR [95% CI]: 1.68 [1.45–1.95]), hepatic encephalopathy (aHR [95% CI]: 1.63 [1.39–1.91]), and hepatic failure (aHR [95% CI]: 1.26 [1.42–1.86]) than nonusers; however, insulin users showed no significant difference in the risk of jaundice (aHR [95% CI]: 0.90 [0.63–1.29]).

Table 2 also displays that insulin users had significantly higher risks of MACE (aHR [95% CI]: 1.41 [1.23–1.62]), stroke (aHR [95% CI]: 1.31 [1.09–1.58]), ischemic heart disease (aHR [95% CI]: 1.36 [1.09–1.71]), and heart failure (aHR [95% CI]: 2.18 [1.70–2.80]) than nonusers.

**Fig. 2:**
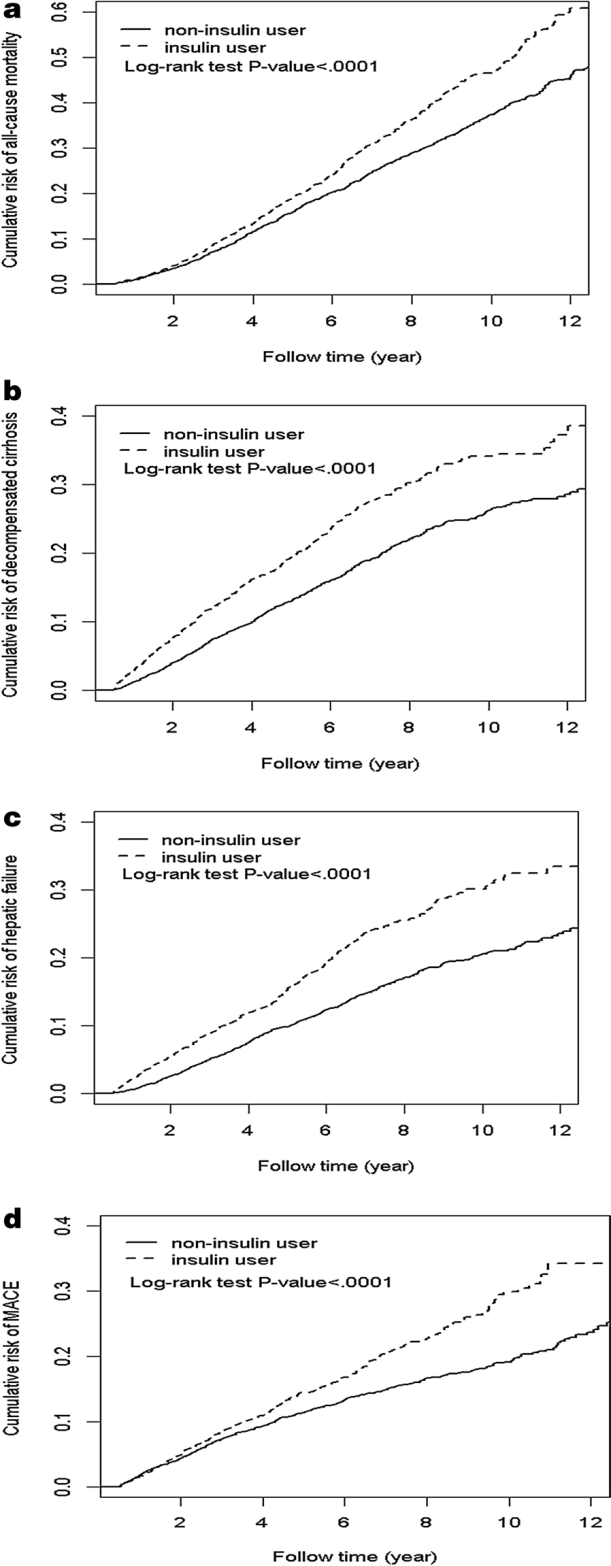
Cumulative incidence curves of four outcomes between insulin users and nonusers

Figure 2 shows the cumulative incidence of all-cause mortality, decompensated cirrhosis, hepatic failure, and MACE of insulin users and nonusers with T2DM and compensated liver cirrhosis.

Insulin users had a higher risk of hypoglycemia (aHR [95% CI]: 3.33 [2.45–4.53]) than nonusers (Table 2).

### Sensitivity analysis

Table 3 presents the results of the sensitivity analysis of all-cause mortality, liver-related outcomes, and MACE, in which persons with hypoglycemia before and during the follow-up periods were excluded. Insulin users showed higher risks of all-cause mortality, HCC, decompensated cirrhosis, hepatic failure, and MACE than nonusers.

**Table 3:** Outcomes of insulin users and matched nonusers after excluding persons with hypoglycemia

Outcomes	Non-insulin users (n = 4026)			Insulin users (n = 1940)			Crude HR (95% CI)	*p* value	Adjusted HR^a^ (95% CI)	*p* value
	Events	PY	IR	Events	PY	IR				
All-cause mortality	951	23,577	4.03	575	11,131	5.17	1.32 (1.19˗1.47)	< 0.0001	1.30 (1.17˗1.44)	< 0.0001
HCC	682	22,041	3.09	386	10,219	3.78	1.21 (1.07˗1.37)	0.003	1.22 (1.08˗1.39)	0.002
MACE	490	21,816	2.25	303	10,152	2.98	1.33 (1.15˗1.54)	< 0.0001	1.37 (1.19˗1.58)	< 0.0001
Stroke	289	22,616	1.28	165	10,620	1.55	1.23 (1.01˗1.48)	0.04	1.25 (1.04˗1.53)	0.02
Ischemic heart disease	188	22,819	0.82	111	10,727	1.03	1.25 (0.99˗1.59)	0.06	1.32 (1.04˗1.68)	0.02
Heart failure	118	23,187	0.51	112	10,841	1.03	2.04 (1.57˗2.64)	< 0.0001	2.12 (1.63˗2.75)	< 0.0001
Decompensated cirrhosis	626	22,411	2.79	432	10,219	4.23	1.50 (1.33˗1.69)	< 0.0001	1.52 (1.34˗1.72)	< 0.0001
Esophageal varicose	41	23,488	0.17	37	11,034	0.34	1.86 (1.20˗2.91)	0.006	1.86 (1.19˗1.92)	0.007
Hepatic ascites	395	22,915	1.72	298	10,518	2.83	1.62 (1.40˗1.89)	< 0.0001	1.65 (1.42˗1.92)	< 0.0001
Hepatic encephalopathy	339	23,105	1.47	265	10,672	2.48	1.67 (1.52˗1.96)	< 0.0001	1.67 (1.42˗1.97)	< 0.0001
Jaundice	101	23,355	0.43	44	11,027	0.40	0.91 (0.64˗1.30)	0.61	0.95 (0.66˗1.36)	0.78
Hepatic failure	481	22,891	2.10	362	10,528	3.44	1.62 (1.42˗1.87)	< 0.0001	1.63 (1.42˗1.88)	< 0.0001

PY, person-years; IR, incidence rate, per 100 person-years; HR, hazard ratio; CI, confidence interval; HCC, hepatocellular carcinoma; MACE, major adverse cardiac event, including stroke, ischemic heart disease, and heart failure

^a^Adjusted for age, sex, index year, DM age, DM duration (years), antihypertensive drugs (ACE inhibitors, ARBs, β-blockers, calcium-channel blockers, diuretics, other antihypertensive), antidiabetic drugs (metformin, sulfonylureas, meglitinides, TZD, α-glucosidase inhibitor, DPP-4 inhibitors), statins, and aspirin, CIC index (0, 1, ≥ 2), DCSI score (0, 1, ≥ 2), HBV and HCV

## Discussion

Our study indicated that in people with T2DM and compensated cirrhosis, insulin users showed higher risks of all-cause mortality, cardiovascular events, HCC, decompensated cirrhosis, hepatic failure, and hypoglycemia than insulin nonusers, even after excluding persons with hypoglycemia.

Insulin treatment is frequently used in persons with diabetes and liver cirrhosis. Elkrief et al. reported that of 348 persons with hepatitis C-related cirrhosis, 62% were on insulin therapy [19]. Gentile et al. found that acarbose significantly improved fasting and postprandial glucose levels in 100 persons with compensated cirrhosis and insulin-treated T2DM [20]. They also compared the metabolic profiles of lispro and regular human insulin in persons with diet-unresponsive T2DM and compensated nonalcoholic liver disease and found that lispro caused lower postprandial glucose levels and hypoglycemic rates [6]. Insulin requirements in persons with liver cirrhosis vary; persons with decompensated cirrhosis may need less insulin compared with persons diagnosed as having compensated cirrhosis [5]. Therefore, insulin therapy in people with liver cirrhosis requires close monitoring of blood glucose levels to avoid the risks of hypoglycemia or hyperglycemia. Our study disclosed that the use of insulin was associated with a significantly higher risk of severe hypoglycemia in persons with compensated cirrhosis compared with oral antidiabetic agents.

People with liver cirrhosis have a 5–10 times higher risk of death than the general population [21], and diabetes can increase their mortality risk [22]. Insulin was reported to be associated with a high risk of mortality in persons with T2DM [23]; our study also showed that among persons with compensated liver cirrhosis, insulin users demonstrated a higher risk of all-cause mortality than insulin nonusers. Although hypoglycemia may increase the risk of death, we observed similar results even after excluding persons with hypoglycemia. Moreover, because insulin users in this study showed increased risks of major cardiovascular events, cirrhotic decompensation, and liver failure, these conditions may also increase the risk of death.

People with liver cirrhosis were reported to have a low prevalence of cardiovascular diseases [24], which may be because of their short life expectancy and low levels of clotting factors in their blood. Coexisting T2DM may increase the risk of cardiovascular diseases; however, their prevalence is still lower than that of general population with T2DM only [24]. Insulin therapy was reported to increase the risk of cardiovascular complications in persons with T2DM [8]. Our study also illustrated that insulin use in persons with compensated liver cirrhosis was associated with a higher risk of MACE, and these hazards persisted even after excluding persons with hypoglycemia. Excess exposure to insulin and hyperinsulinemia are thought to increase basal insulin signaling, which can contribute to insulin resistance and cause atherosclerosis [25].

T2DM [5, 19, 26] and suboptimal glycemic levels [26] in persons with liver cirrhosis were reported to increase the risks of liver-related complications. However, the favorable impact of optimal glycemic management in persons with liver cirrhosis has not been demonstrated yet. Our study compared the progression of cirrhotic complications between insulin users and nonusers with compensated cirrhosis and observed that insulin users seemed to have higher risks of variceal bleeding, ascites, hepatic encephalopathy, and hepatic failure than insulin nonusers. Insulin stimulates adrenergic hormones and releases endothelin-1 [27]. It was reported to have vasoconstrictor effects on isolated arterioles [28], which may increase systemic vascular resistance and portal pressure. Cirrhosis can aggravate insulin resistance and disturb the molecular mechanisms of insulin on hepatocytes. Exogenous insulin and consequent hyperinsulinemia may activate some signaling molecules (such as PHLPP1 and Grb14) and influence hepatocyte apoptosis [29, 30]. These factors may exacerbate the progression of liver cirrhosis and hepatic failure.

HCC occurs primarily in persons with cirrhosis, and diabetes can exacerbate this risk [5]. The use of insulin was reported to increase the risk of HCC [10, 26]; our study supports this finding because our results showed that insulin users had a higher risk of HCC than insulin nonusers. Through the activation of the insulin-like growth factor signaling pathway, exogenous insulin and hyperinsulinemia may accelerate hepatocarcinogenesis in persons with liver cirrhosis.

This study has some limitations. First, this was a nationwide cohort study using a sample of Chinese ethnicity only; therefore, the results cannot be generalized to other ethnicities. Second, the administrative claims dataset does not have information on body weight, physical activity, alcohol consumption, and cigarette smoking. It does not contain data on blood biochemical and hemoglobin A1C results, which are used to assess the severity of liver cirrhosis and the treatment situation of T2DM. Instead, we used clinical diagnoses to divide persons into those with compensated and decompensated liver cirrhosis and used DCSI and diabetes duration to distinguish the severity of T2DM. We performed propensity score matching to balance critical variables between insulin users and nonusers to maximally reduce the bias from known confounders. However, the above mentioned unmeasured factors may affect our results. Third, due to no linkage to the National Death Registry, the definition of death in this study includes patients no longer coverage by NHI after discharge from critical illness; which may overestimate the incidence of mortality. Fourth, because the number of insulin pens is counted instead of units of insulin in insulin prescription in our health system, we cannot accurately calculate the doses of insulin used. The patients’ adherence to prescribed insulin injections or oral antidiabetic drugs also cannot be adequately measured using this health insurance database. Moreover, physicians may choose insulin therapy according to the severity of patients, this cofounding by indication also needs to be noted. Finally, a cohort study is always subject to some inevitable bias, and randomized controlled studies are warranted to verify our results.

## Conclusion

Although insulin is the recommended treatment for persons with T2DM and liver cirrhosis, few clinical studies have evaluated its long-term effects and safety. In this retrospective cohort study, insulin use in people with T2DM and compensated cirrhosis was associated with higher risks of hypoglycemia, cardiovascular events, liver-related complications, and mortality than insulin nonusers. Therefore, in persons with compensated liver cirrhosis, the use of insulin may require special attention.

## Data Availability

Data are available from the National Health Insurance Research Database (NHIRD) published by Taiwan National Health Insurance (NHI) Bureau. The data utilized in this study cannot be made available in the paper, the supplemental files, or in a public repository due to the ‘‘Personal Information Protection Act’’ executed by Taiwan’s government, starting from 2012. Requests for data can be sent as a formal proposal to the NHIRD (http://nhird.nhri.org.tw) or by email to nhird@nhri.org.tw.

## Abbreviations

T2DM: Type 2 diabetes mellitus
HBV: Hepatitis B virus
HCV: Hepatitis C virus
HCC: Hepatocellular carcinoma
CCI: Charlson comorbidity index
DCSI: Diabetes complication severity index
MACE: Major adverse cardiovascular events
